# Comment: Respecifying Emotional Influence

**DOI:** 10.1177/1754073916669597

**Published:** 2017-06-15

**Authors:** Brian Parkinson

**Affiliations:** Department of Experimental Psychology, University of Oxford, UK

**Keywords:** emotional influence, relation alignment, social appraisal, social referencing

## Abstract

To what extent does the level of overlap between social appraisal and social referencing depend upon the particular definitions adopted when following different research agendas? I argue that processes of both kinds fall under the more inclusive heading of relation alignment. Relation alignment also covers emotional influence that is not mediated by the communication of appraisal. Similarities, interdependences, and distinctions between these various relation-alignment processes warrant further investigation.

Are social appraisal and social referencing the same thing? Is there underlying commonality in the kinds of emotional influence implied by both concepts? Or is social referencing just one example of a number of possible social appraisal processes? Answers to these questions obviously depend on our definitions of “social appraisal” and “social referencing” because adopting a more restrictive or inclusive formulation of either construct can tip the balance of argument in one or the other direction. For example, a relatively broad concept of social appraisal is more likely to encompass a relatively narrow concept of social referencing than vice versa. But is there something deeper at stake; something that goes beyond semantics and gets to the bottom of how emotions regulate other people’s behaviour across social development and during real-time interactions between adults?

Three decades back, similar considerations surrounded the affective primacy controversy (Lazarus, 1984; Zajonc, 1984). Whether emotion always depends on cognition is another question whose answer hinges on how inclusively the key concepts are formulated (e.g., Leventhal & Scherer, 1987; Parkinson & Manstead, 1992). Emotion without cognition is clearly possible if “emotion” is defined broadly enough and “cognition” is defined narrowly enough. However, definitional respecification of this kind leaves more fundamental questions about what kinds of “emotion” depend on what kinds of “cognition” unanswered (Leventhal & Scherer, 1987). In the affective primacy controversy, moving beyond definitional disputes involved addressing these more specific questions. Arguably, the burgeoning study of interpersonal emotional influence processes should follow a similar trajectory.

Different researchers have certainly used the terms “social appraisal” and “social referencing” in a variety of overlapping senses. Although both concepts consistently refer to interactions in which one person’s emotion influences another person’s evaluative orientation to an object or event, these interactions can involve a range of processes, and these processes can serve a range of functions. The extent to which the processes and functions need to be distinguished depends on the agenda guiding the research in question. When the theoretical goal is to characterize the spectrum of interpersonal processes whereby people acquire information about the affective significance of events, it makes sense to make corresponding distinctions (e.g., Clément & Dukes, 2017). When the theoretical goal is to uncover unifying principles underlying the development of interpersonal emotional influence, continuities rather than discontinuities start to seem more important (Walle, Reschke, & Knothe, 2017).

My research (e.g., Parkinson, Simons, & Niven, 2016) mainly focuses on real-time interactions in which adult “knowers” and “learners” repeatedly switch roles and where influence operates dynamically and bidirectionally. In these contexts, each person’s attention can shift rapidly from a referential object to the other person, or to the relation between self and other, or between self or other and object. For example, a wife expresses worry about her ability to cope with a current concern, and her husband may orient at different moments to the concern, his wife, her orientation to him, and to the concern (e.g., Parkinson & Simons, 2012). Each partner may strategically regulate some aspects of their emotional orientation, while other (often simultaneous) processes of mutual adjustment operate at less explicit levels (e.g., Parkinson & Simons, 2009).

I use the flexible term “relation alignment” (e.g., Parkinson, 2008) to capture the variety of emotional influence processes operating during these interactions. Relation alignment includes social appraisal along with other forms of interpersonal adjustment that do not depend on explicit communication or transfer of appraisal information. For me, social referencing is an even more specific concept covering a particular form of interaction in which one person actively seeks out clarificatory evaluative information from another person. This evaluative information then affects the second person’s behaviour by a process of social appraisal. In other words, both social appraisal and social referencing imply interpersonal information transfer and communication of emotional meaning, whereas relation alignment can operate prior to, and as part of, the production of emotional meaning.

More basic forms of relation alignment also seem to precede the acquisition of social appraisal and social referencing in developing children. Infants adjust to (e.g., Stern, Hofer, Haft, & Dore, 1985) and actively operate on (e.g., Reddy, 2008) caregivers’ emotions before they begin to represent the relational meaning of those emotions in terms of appraisal (Parkinson, 2007). Indeed, some of the earliest forms of infant–caregiver interaction involve regulation of direct relations between interactants rather than their respective orientations towards any referential object. Social referencing probably depends on subsequent co-ordination of this more directly interpersonal relation alignment with the development of shared attention towards objects.

Despite its potential reducibility to a definitional dispute, the old affective primacy debate at least served to draw attention to emotion-related phenomena that did not depend on prior generation of explicit meaning. Similarly, I want to argue that whatever forms of “cognition,” “appraisal,” or “social referencing” characterize the earliest and most basic instances of relation alignment, they do not necessarily involve ostension or the active extraction of appraisal information. Mutual orientations can adjust to one another, and their interpersonal consolidation at some point produces emergent emotional meanings (e.g., Fogel, 1993), which need not be registered as such by either party to the exchange.
